# Hypoperfusion Intensity Ratio as an Independent Predictor of Functional Outcome After Mechanical Thrombectomy for Large Vessel Occlusion Stroke

**DOI:** 10.3390/medicina62040731

**Published:** 2026-04-11

**Authors:** Dagnija Grabovska, Arturs Balodis, Arvīds Bušs, Madara Ratniece, Roberts Šamanskis, Evija Miglāne, Kārlis Kupčs, Kristaps Jurjāns, Arta Grosmane, Sigita Zālīte, Maija Radziņa

**Affiliations:** 1 Institute of Diagnostic Radiology, Pauls Stradins Clinical University Hospital, LV-1002 Riga, Latvia; 2 Faculty of Medicine and Life Sciences, University of Latvia, LV-1586 Riga, Latvia; 3 Department of Radiology, Riga Stradiņš University, LV-1007 Riga, Latvia; 4 Faculty of Medicine, Riga Stradins University, LV-1007 Riga, Latvia; 5 Department of Neurology, Pauls Stradins Clinical University Hospital, LV-1002 Riga, Latvia; 6 Department of Neurology and Neurosurgery, Riga Stradiņš University, LV-1007 Riga, Latvia

**Keywords:** stroke, hypoperfusion intensity ratio, collateral circulation, CT perfusion, mechanical thrombectomy, artificial intelligence

## Abstract

*Background and Objectives*: Acute ischemic stroke (AIS) caused by large vessel occlusion (LVO) remains a major cause of disability and mortality. Mechanical thrombectomy (MT) improves outcomes, but recovery varies. This study assessed the prognostic value of hypoperfusion intensity ratio (HIR), collateral circulation, and other clinical/imaging factors. *Materials and Methods*: This retrospective cohort study included 96 LVO patients treated with MT with or without intravenous thrombolysis (IVT) between 2020 and 2024 at a tertiary hospital. Inclusion required multimodal CT (CT, CTA, CTP) and clinical data (NIHSS, mRS). HIR, core volume, CBV index, mismatch ratio, and collateral status were evaluated using artificial intelligence (AI)-based software. Univariate/multivariate logistic regression identified predictors of poor outcome (mRS > 3 at 90 days). *Results*: Lower HIR (<0.5) and good collaterals were associated with favourable outcomes (*p* < 0.001). Multivariate analysis identified HIR, initial NIHSS, and procedure duration as independent predictors of poor outcome. CTP-derived core volume, cerebral blood volume index, and mismatch ratio were also significant predictors. ROC analysis showed the highest AUC for core volume (0.810). Diabetes mellitus was associated with a worse prognosis compared to other clinical factors. *Conclusions*: HIR and collateral status are independent predictors of functional recovery after MT. CTP-derived core volume and CBV index have strong prognostic value. AI-based perfusion analysis supports patient selection and risk stratification.

## 1. Introduction

Stroke is a leading cause of death and long-term disability worldwide and imposes a substantial clinical and economic burden on patients, families and healthcare systems. Large vessel occlusion (LVO) of the anterior circulation accounts for a considerable proportion of disabling strokes and is associated with high mortality and poor functional recovery if left untreated [1,2,3,4].

Mechanical thrombectomy (MT), often combined with intravenous thrombolysis (IVT), has become the standard-of-care for LVO stroke and markedly improves outcomes when performed early [5,6]. Nevertheless, a substantial proportion of patients remain functionally dependent despite successful reperfusion. Prognosis is influenced by multiple clinical and imaging factors, including baseline neurological deficit, infarct core volume, collateral status, time to reperfusion and procedural characteristics. Reliable imaging biomarkers are therefore needed to refine patient selection, individualise treatment and better estimate the likelihood of functional recovery.

CT perfusion (CTP) and artificial intelligence (AI)-based perfusion software provide rapid quantitative maps of infarct core, hypoperfused tissue and, indirectly, collateral status. Among these parameters, the hypoperfusion intensity ratio (HIR)—defined as the volume of tissue with Tmax > 10 s divided by the volume with Tmax > 6 s—has emerged as a promising biomarker that reflects the severity of hypoperfusion and acts as a surrogate of collateral flow [7,8,9,10,11]. Several cohort studies have shown that lower HIR values are associated with more favourable collateral circulation, smaller final infarct volumes, slower infarct growth and a higher likelihood of functional independence, whereas higher HIR values correlate with poor collaterals, larger core volumes, futile recanalization and an increased risk of haemorrhagic transformation after mechanical thrombectomy [7,8,9,11,12,13,14]. In parallel, other CTP-derived biomarkers such as relative cerebral blood volume and critical hypoperfusion volumes also demonstrate prognostic value, suggesting that HIR should be viewed as a complementary, rather than stand-alone, marker within a multiparametric perfusion assessment [10,14,15]. However, most available data come from observational studies and reviews based on highly selected trial or registry populations and specific automated software platforms, and the additional prognostic value of HIR over conventional clinical and imaging variables in routine mechanical thrombectomy practice remains incompletely defined [10,12,13,14].

Therefore, the aim of this single-centre retrospective cohort study was to assess whether baseline HIR derived from AI-based CTP is independently associated with 90-day functional outcome in patients with anterior circulation LVO treated with MT (±IVT), after adjustment for established clinical, imaging and procedural predictors. Secondary objectives were to (1) evaluate the relationship between HIR, collateral status and haemorrhagic complications, and (2) compare the prognostic performance of HIR with other automated CTP parameters, including infarct core volume, CBV index and mismatch ratio.

## 2. Materials and Methods

To evaluate the prognostic value of the HIR and collateral cerebral circulation in predicting functional outcomes in patients with acute stroke of the LVO, a retrospective cohort study was conducted. The study included 96 patients aged 18 to 95 years who were treated in the Stroke Unit of the Neurology Clinic at a tertiary university hospital between 2020 and 2024 and who underwent MT with or without intravenous thrombolysis (IVT).

Inclusion criteria comprised patients with complete CT perfusion (CTP) imaging data and documented neurological evaluations both at the time of initiation of treatment and 90 days after MT. Exclusion criteria included incomplete or low quality radiological imaging, unrelated cerebral pathologies (e.g., brain tumours, prior large infarcts), and missing clinical data such as National Institutes of Health Stroke Scale (NIHSS) or modified Rankin scale (mRS) scores. Overall, 103 patients treated during the study period were screened, of whom 96 met the predefined eligibility criteria and were included in the final analysis; 4 were excluded because of incomplete or inadequate imaging, 3 because of missing clinical follow-up data.

**Figure 1:**
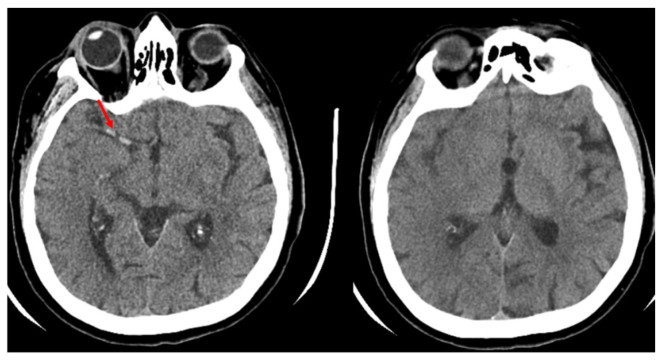
Patient case example: Evaluation of multimodal CT imaging. A 73-year-old patient was admitted with clinical signs of acute ischemic stroke. The initial NIHSS score was 21. The axial images of the non-enhanced head CT show a loss of grey-white matter differentiation in an extensive region within the territory of the right middle cerebral artery (MCA). The ASPECTS was 3 points. In addition, a thrombus is visible in the proximal segment of the right MCA, indicated by the hyperdense artery sign (marked with an arrow).

The following imaging modalities were analysed: non-contrast CT (NCCT), CT angiography (CTA), and CT perfusion (CTP), all performed using a 64-slice CT scanner (Revolution Maxima, GE Healthcare, Chicago, IL, USA). In the initial NCCT, the Alberta Stroke Program Early CT Score (ASPECTS) was used to assess the extent of early ischemic changes. ASPECTS was evaluated using standardised criteria by scoring ischemic changes in ten specific brain regions (Figure 1), providing a baseline estimate of the infarct core volume.

**Figure 2:**
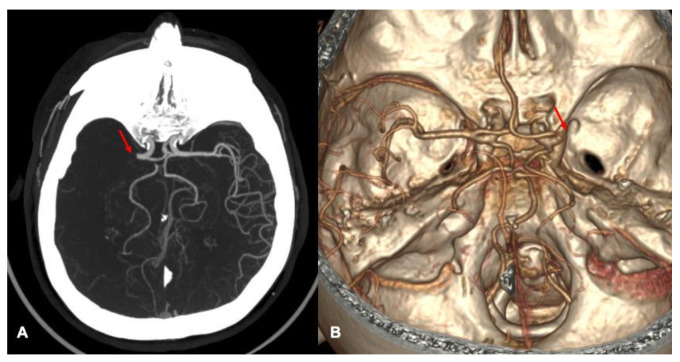
Patient case example: Evaluation of multimodal CT imaging. A 73-year-old female patient’s CTA imaging revealed an occlusion in the proximal segment of the right MCA (M1 segment), with malignant collaterals (0 points). (**A**) Maximum intensity projection (MIP) image showing the occlusion of the M1 segment of the right MCA, indicated by an arrow. (**B**) 3D CTA reconstruction highlighting the M1 segment occlusion (indicated by an arrow), providing enhanced visualisation of the location and extent of the thrombus.

Subsequently, collateral cerebral circulation was assessed in CTA using a three-point grading scale, which evaluates blood flow in the territory distal to the occlusion: 0 points—malignant collaterals, 1 point—poor collateral circulation, 2 points—good collateral circulation (Figure 2).

**Figure 3:**
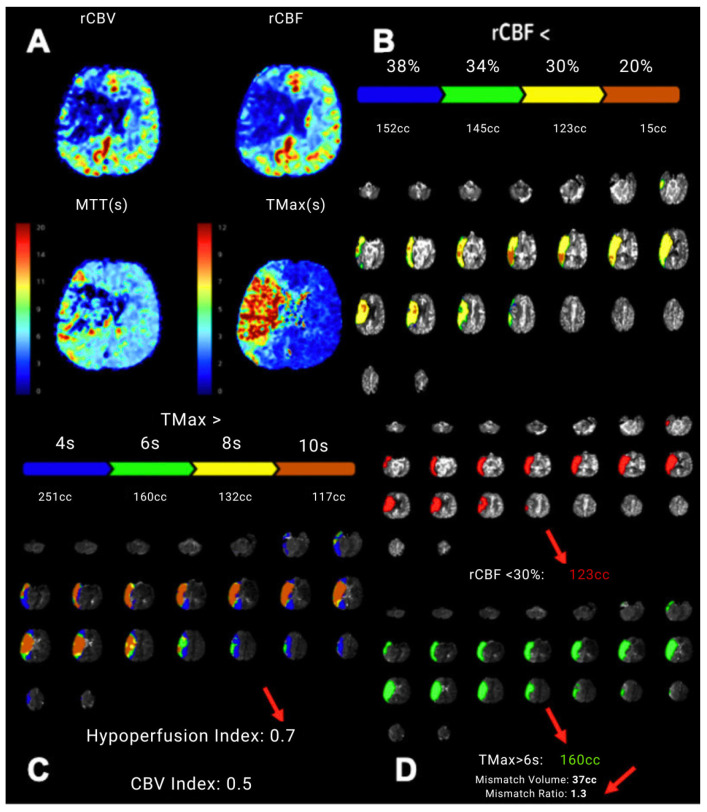
Patient case example: Evaluation of multimodal CT imaging. In the same patient: (**A**) CTP imaging revealed a large penumbra in the right MCA territory, involving the lateral portions of the frontal, parietal, and temporal lobes. Within this area, the core was identified in the right frontal and parietal lobes, the insula, and the right caudate nucleus. (**B**–**D**): Automated measurements were obtained using the AI-based software Viz CTP, which calculated the core and penumbra volumes, mismatch volume, mismatch ratio, and the HIR value (indicated by arrows).

CTP image analysis was performed using artificial intelligence software Viz CTP (Version: 1.9; Viz.ai; San Francisco, CA, USA), which automatically calculated the volumes of the ischemic core and penumbra, the core/penumbra mismatch ratio, as well as the HIR (Figure 3).

**Figure 4:**
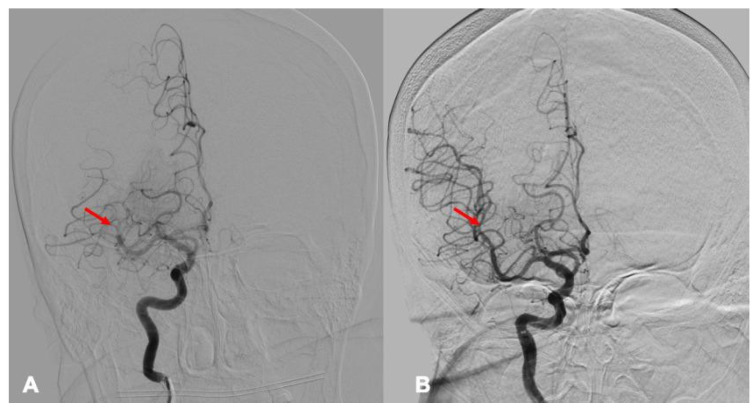
Patient case example: Mechanical thrombectomy evaluation. In the same patient, digital subtraction angiography (DSA) did not reveal visible occlusion in the right MCA M1 segment, previously identified on CTA, indicating spontaneous recanalization. However, an occlusion in the M2 segment (**A**, red arrow) was detected and treated with mechanical thrombectomy. A total of four passes were performed, achieving a reperfusion level of mTICI 2C (**B**). The duration of the procedure was 43 min and the post-procedure NIHSS score was 1 point.

In addition to the analysis of HIR and collateral circulation, the following parameters were also evaluated: duration of mechanical thrombectomy (in minutes), success of recanalization based on the modified Thrombolysis in Cerebral Infarction (mTICI) scale, time from the onset of symptoms to the initiation of MT (Figure 4), the neurological status of the patient evaluated using the NIHSS, and functional outcome at 90 days using the mRS. Mechanical thrombectomy procedures were performed using a biplane angiography system (Artis Zee Biplane, Siemens Healthineers, Erlangen, Germany).

In addition, symptomatic intracerebral haemorrhage (sICH) within 24 h after treatment was defined according to the Safe Implementation of Thrombolysis in Stroke-Monitoring Study (SITS-MOST) criteria as a parenchymal haemorrhage type 2 (PH2) on follow-up CT associated with a neurological deterioration of ≥4 points on the NIHSS from baseline or from the lowest value within the first 24 h, or resulting in death.

The variables described previously were analysed to evaluate their impact on functional recovery and their association with the hypoperfusion intensity ratio (HIR), automated CTP measurements, and other clinical and radiological parameters.

### Statistical Analysis

Data were compiled and analysed using MS Excel and IBM SPSS Statistics (version 31.0.1.0) software. All included variables were tested for type consistency (categorical or numerical) and normality was assessed prior to testing using the Shapiro–Wilk test, supplemented by visual inspection of histograms and Q–Q plots.

Long-term functional outcomes were classified as favourable (mRS 0–3) or unfavourable (mRS 4-6) 90 days after the procedure. This cut-off was chosen because our cohort consisted predominantly of elderly patients with severe baseline deficits.

Descriptive statistics were used to summarise demographic and clinical characteristics. Normally distributed continuous variables (e.g., age) are presented as mean ± standard deviation (SD), whereas non-normally distributed continuous variables and ordinal variables (e.g., NIHSS and mRS scores) are presented as median (interquartile range, IQR). Categorical variables (e.g., sex and comorbidities) are reported as absolute frequencies (*n*) and percentages (%).

For comparisons of non-normally distributed continuous variables between two groups, the Mann–Whitney U test was used. Differences in categorical variables were evaluated using Fisher’s exact test, Pearson’s Chi-square test or Monte Carlo simulation, depending on the data structure. The effect sizes were calculated using Phi and Cramér’s V coefficients.

Furthermore, a univariate analysis was performed to identify potentially prognostic automated CT perfusion variables, including core volume (cerebral blood flow (CBF) <30%), mismatch ratio, cerebral blood volume (CBV) index and mismatch volume.

To identify independent predictors of functional outcome, a binary logistic regression analysis was performed. Because of the limited sample size and the need to minimise model overfitting, variable selection for multivariable modelling was restricted to factors showing associations in univariable analysis. The results were expressed as odds ratios (ORs) with 95% confidence intervals (CI) and *p*-values. Two separate regression models were developed, one excluding HIR and another including HIR, to assess their independent prognostic value beyond other relevant predictors.

In addition, receiver operating characteristic (ROC) analysis was used to evaluate the discriminative performance of the multivariable logistic regression models and of individual automated CTP parameters. ROC curves for the prediction of poor functional outcome were constructed using the predicted probabilities from each model and the continuous values of core volume (CBF < 30%), mismatch ratio and CBV index.

A *p*-value < 0.05 was considered statistically significant.

## 3. Results

### 3.1. General Patient Data

The study included 96 patients, of whom 62.5% (*n* = 60) were female and 37.5% (*n* = 36) were male. The age range was 37 to 93 years, with a mean age of 75.8 years (SD = 9.6). The main demographic and clinical characteristics of the patients are presented in Table 1.

The baseline neurological deficit, assessed using the NIHSS, was 16.0 (IQR 11.8–19.0). Initial functional status, measured by the mRS, was 5.0 (IQR 5.0–5.0). At 90 days after the intervention, the median mRS was 5.0 (IQR 1.8–6.0).

### 3.2. Factors Associated with the Functional Outcome

Based on the binary variable analysis, several statistically significant factors were identified as associated with functional outcome 90 days after mechanical thrombectomy, as measured by mRS.

Collateral circulation quality, evaluated by CTA, was strongly associated with functional outcome (*p* < 0.001), with Cramér’s V = 0.519, indicating a strong positive correlation. Patients with good collateral supply were significantly more likely to achieve a favourable outcome (mRS ≤ 3).

The number of thrombectomy passes was also a significant predictor (Monte Carlo *p* < 0.001; Fisher’s exact test *p* < 0.05; Cramér’s V = 0.376), indicating a moderate positive correlation; fewer passes were associated with better clinical results.

Diabetes mellitus was associated with worse outcomes (*p* < 0.05; Phi = −0.295), showing a statistically significant negative correlation.

Other factors, such as sex, hypertension, dyslipidaemia, smoking, atrial fibrillation, coronary artery disease, chronic heart failure, previous stroke or haemorrhage, and DVT/PE, did not show statistically significant associations (*p* > 0.05).

Using the Mann–Whitney U test, several univariate predictors significantly associated with mRS outcomes at 90 days were identified. Shorter duration of the procedure was associated with favourable outcomes (*p* < 0.001). Lower initial NIHSS scores were found in the good outcome group (*p* = 0.004). Lower HIR values were observed in patients with good recovery (*p* < 0.001). Higher ASPECTSs on baseline CT, perfusion core ASPECTS, and follow-up CT were associated with good outcomes (*p* < 0.05, *p* < 0.001, *p* < 0.001, respectively). Better mRS scores prior to stroke were associated with favourable outcomes (*p* < 0.05).

However, the following variables were not statistically significant. Age showed no significant differences between the groups (*p* > 0.05), suggesting that age alone is not a decisive factor in outcome. Time to recanalization, while clinically relevant, was not statistically significant in this analysis (*p* > 0.05). Platelet count showed no significant association with outcome.

### 3.3. Logistic Regression Analysis

Univariable and multivariable logistic regression results are summarised in Table 2. A binary logistic regression was performed to identify independent predictors of an unfavourable functional outcome (mRS > 3) 90 days after stroke in patients treated with mechanical thrombectomy. Two models were constructed, one excluding HIR and one including HIR, in order to assess its independent prognostic contribution.

**Figure 5:**
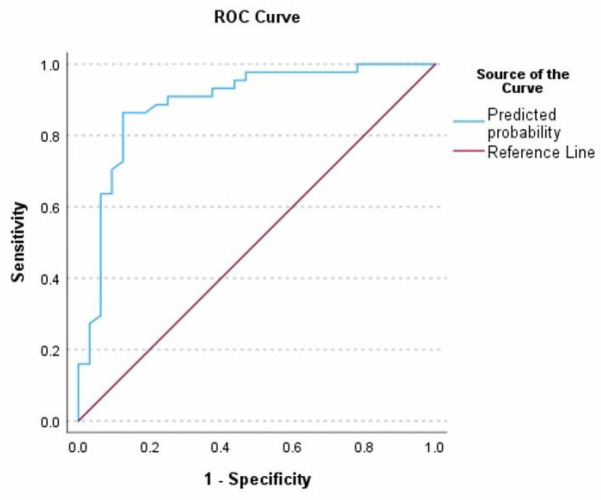
Receiver operating characteristic (ROC) for poor functional outcome (mRS > 3) at 90 days after stroke in patients treated with mechanical thrombectomy—without inclusion of HIR. The following parameters were found to be statistically significant predictors of poor outcome: (1) Longer duration of the procedure (*p* < 0.005); (2) Higher baseline NIHSS score (*p* < 0.05); (3) Poor collateral circulation (*p* < 0.05); (4) Presence of diabetes mellitus (*p* < 0.05). These variables remained independently associated with worse outcomes even after controlling for potential confounders. The model highlights the importance of timely intervention, initial stroke severity, collateral status, and metabolic comorbidities in predicting recovery after thrombectomy.

In the model without HIR, four independent predictors of poor outcome were identified. Regarding the duration of the procedure, each additional minute increased the risk of a poor outcome by 5.8%. Regarding the initial NIHSS score, each additional point increased the risk by 17.5%. Good collateral circulation is associated with a 91.4% lower risk of poor outcome or 11.6 times higher odds of favourable recovery. Absence of diabetes mellitus is associated with an 86.9% lower risk of poor outcome or 7.6 times higher odds of favourable outcome. See Figure 5 for a visual summary of the regression model.

**Figure 6:**
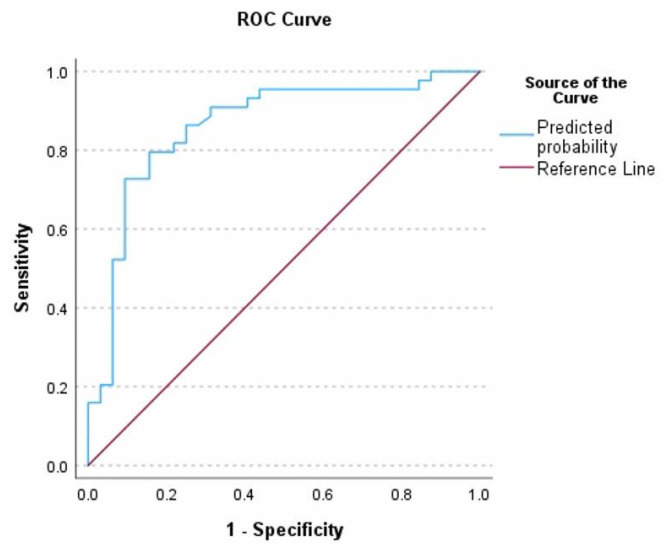
Receiver operating characteristic (ROC) for poor functional outcome (mRS > 3) at 90 days after stroke in patients treated with mechanical thrombectomy—including HIR. The analysis confirmed the following statistically significant predictors of an unfavourable outcome. (1) Duration of the procedure: Each additional minute increased the risk by 5.1% (*p* < 0.005; Exp(B) = 1.051; 95% CI: 1.016–1.087); (2) Initial NIHSS score: Each additional point increased the risk by 18.4% (*p* < 0.05; Exp(B) = 1.184; 95% CI: 1.034–1.356); (3) HIR: For every 0.1 unit increase, the risk of poor outcomes increased by 47.6% (*p* < 0.05; Exp(B) = 1.476; 95% CI: 1.077–2.027). These findings demonstrate that HIR is an independent and clinically relevant predictor of long-term functional outcome after mechanical thrombectomy.

In the second model, which included the hypoperfusion intensity ratio (HIR), the results showed a similar trend. The duration of the procedure and the initial NIHSS score remained statistically significant predictors of a poor outcome. Each additional minute of procedure time was associated with a 5.1% increase in the risk of poor outcome. Each additional point in the NIHSS was associated with an 18.4% increase in risk. Importantly, HIR emerged as an independent and significant predictor of a worse prognosis. For every 0.1 unit increase in HIR, the probability of a poor outcome increased by 47.6%. See Figure 6 for a visual representation of the model.

When comparing the predictive performance of both models, high prognostic precision was observed in each. The model without HIR achieved an AUC of 0.861, with a sensitivity of 72%, a specificity of 90.9%, and an overall accuracy of 82.9%. The HIR model showed an AUC of 0.890, a sensitivity of 75%, a specificity of 81.8% and an overall accuracy of 78.9%. These findings indicate that factors such as shorter procedure time, lower initial NIHSS, better collateral circulation, and HIR are clinically significant predictors of stroke outcomes. In addition, the absence of diabetes mellitus emerged as a favourable prognostic indicator. Although the inclusion of HIR slightly reduced overall precision, it provided independent prognostic value as an additional perfusion biomarker, supporting its role in improving patient selection criteria and optimising treatment strategies in acute ischemic stroke management.

### 3.4. Evaluation of Symptomatic Intracerebral Haemorrhage

The incidence of symptomatic intracerebral haemorrhage, defined according to SITS-MOST criteria, in the study cohort was low, occurring in 9 out of 96 patients (9.4%). To assess possible differences between patients with and without sICH, a comparative analysis was performed. However, no statistically significant differences were found in any of the clinical, procedural, or radiological parameters (*p* > 0.05). Due to the small number of patients, the statistical power of the analysis was limited and the findings should be interpreted as descriptive, without the ability to draw firm conclusions about influencing factors.

### 3.5. Evaluation of Automated CTP Measurements

To identify automated CT perfusion (CTP) parameters associated with functional recovery after acute ischemic stroke, a nonparametric univariate analysis was performed using the Mann–Whitney U test. Three variables showed statistically significant correlations with the results of the mRS at 90 days: core volume (CBF < 30%) (*p* < 0.001); mismatch ratio (*p* < 0.001); CBV index (*p* < 0.001).

**Figure 7:**
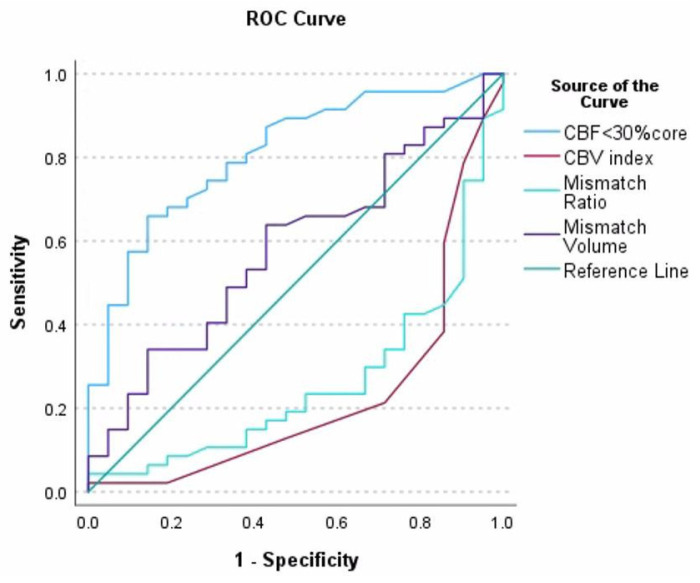
Receiver operating characteristic (ROC) based on automated CTP measurements. Among the variables tested, the core volume (CBF < 30%), CBV index and the mismatch ratio were included based on their statistically significant associations in the univariate analysis. The model identified the following independent predictors of poor outcome. (1) Core volume (CBF < 30%) was a significant predictor, where each 1 mL increase in infarct core volume was associated with an increase in the odds of poor outcome (Exp(B), confidence interval, and *p*-value to be specified depending on results). (2) The lower CBV index was also associated with worse outcomes, suggesting reduced tissue viability. (3) The mismatch ratio, although significant in the univariate analysis, showed a reduced predictive strength in the multivariate model, probably due to collinearity with other perfusion parameters.

These findings suggest that larger infarct core volumes, lower cerebral blood volume (CBV) indices, and greater perfusion mismatch ratios are significantly associated with worse functional outcomes after stroke. In contrast, mismatch volume did not show a statistically significant relationship with mRS (*p* = 0.072), indicating its limited prognostic value within this study population. To evaluate the predictive accuracy of the identified parameters for unfavourable outcomes, receiver operating characteristic (ROC) analysis was performed, further confirming their diagnostic utility (Figure 7). The core volume (CBF < 30%) demonstrated the highest discriminative power with an AUC = 0.810. The optimal threshold was 19 mL, producing a sensitivity of 72.9% and a specificity of 72.0%. The CBV index had an AUC = 0.765, with a threshold of 0.75, sensitivity 71.4% and specificity 74.5%. The mismatch ratio achieved an AUC of 0.748, with an optimal cutoff value of 11.0, a high sensitivity of 81.8%, but a lower specificity of 50.0%.

Overall, the results indicate that core volume and the CBV index are particularly important prognostic biomarkers, which may aid in predicting functional recovery in patients undergoing mechanical thrombectomy for large vessel occlusion (LVO) stroke.

## 4. Discussion

This study explored several key prognostic factors that influence treatment outcomes in patients with acute ischemic stroke due to large vessel occlusion (LVO) who underwent MT. Based on a literature review and clinical data analysis, the study highlights two critical elements, the hypoperfusion intensity ratio (HIR) and collateral circulation, evaluating their impact on functional outcomes after stroke and the potential of AI to automate the assessment of these variables.

HIR has emerged as a valuable prognostic biomarker. It is one of the most accurate quantitative indicators of hypoperfusion severity in ischemic brain tissue. Previous studies have shown that lower HIR values correlate with better collateral circulation, smaller infarct core volumes, and improved functional recovery (as measured by mRS at 90 days) [16,17]. In addition, recent studies have shown a correlation between HIR and collateral flow assessed by digital subtraction angiography (DSA). A threshold of HIR < 0.4 has been identified as optimal for predicting the favourable collateral status on DSA. However, it is important to note that the optimal threshold to detect good collaterals may differ between imaging modalities, for example, multiphase CT angiography (mCTA) versus DSA, necessitating modality-specific validation [9]. Most current research relies on the RAPID software platform, but other automated systems such as AIDOC, Viz CTP, and Syngo.via are also capable of calculating HIR. Although these platforms generally yield comparable results, there is insufficient evidence to confirm that HIR values from different software solutions consistently correlate with collateral flow assessment. Therefore, more studies are needed to evaluate the relationship between HIR and collateral status and establish clinically applicable threshold values to predict favourable collateral flow on different imaging platforms [9,10]. These findings are consistent with the hypothesis of this study and the previous literature, which considers HIR values < 0.4–0.5 to be favourable for predicting reperfusion outcomes.

HIR appears to provide complementary prognostic information rather than replace established predictors of outcome. While baseline stroke severity, infarct extent, and collateral status remain central to clinical decision-making, HIR may reflect an additional aspect of ischemic pathophysiology, namely the severity of hypoperfusion and the efficiency of collateral circulation at the tissue level. In practical terms, HIR may also simplify the interpretation of complex perfusion data by condensing the severity of critical hypoperfusion into a single automated and quantitative parameter, rather than relying on the separate assessment of multiple perfusion-derived measurements. In our cohort, HIR remained independently associated with 90-day functional outcome in the multivariable model together with procedure duration and baseline NIHSS, suggesting that it may add incremental prognostic value within a multimodal assessment framework. However, this added value should be interpreted cautiously given the retrospective single-centre design and limited sample size.

In addition, HIR serves as a potentially objective indicator of collateral cerebral blood flow and can serve as a complementary and potentially more objective adjunct to traditional collateral assessment methods based on CTA. Unlike subjective scoring systems such as the ASITN-CS scale, the HIR is automated, quantitative, and reproducible, providing fast and reliable information on cerebral perfusion status. Although the correlation between HIR and ASITN-CS is weak, it remains statistically significant, and higher HIR values consistently indicate poorer collateral status. This underlines HIR’s utility in predicting reperfusion potential and guiding treatment strategies [5].

HIR values are also influenced by individual patient characteristics, such as body mass index (BMI) and age. Studies show that patients with a lower BMI and advanced age are more likely to have poor collateral circulation, possibly due to age-related vascular decline. These factors should be considered when interpreting HIR and planning treatment, highlighting the need for a patient-specific evaluation in clinical practice [18].

The CT perfusion maps used to calculate HIR allow visualisation and quantification of the infarct core-to-penumbra ratio, delineating the region between irreversibly damaged and potentially salvageable brain tissue. HIR not only enables a clear demarcation of these zones but also provides a reliable quantitative assessment. This is especially valuable in cases where recanalization fails, as HIR offers the most accurate prediction of the final infarct volume. Favourable HIR profiles are associated with good collateral flow, smaller initial core volumes, larger penumbras, and better functional outcomes after treatment [19,20].

HIR appears to have potential clinical applicability and may be incorporated into future multimodal prediction frameworks for thrombectomy patient selection. However, HIR values can be influenced by both technological factors (e.g., perfusion protocols, software variability) and biological factors, including age, BMI, and individual cerebrovascular architecture [16]. Thus, while HIR is an objective and automatable parameter, its interpretation should still consider individual clinical contexts.

The analysis of automated CTP parameters further enriched the interpretation of imaging data. Infarct core volume (CBF < 30%), CBV index, and mismatch ratio were significantly associated with functional outcome at 90 days, while mismatch volume was not. Among these, core volume showed the highest discriminative ability in ROC analysis (AUC = 0.810), underscoring its importance for risk stratification. CBV index and mismatch ratio also demonstrated moderate prognostic value.

Although the Alberta Stroke Program Early CT Score (ASPECTS) is widely used to assess acute stroke severity and predict outcomes [21,22], growing clinical evidence highlights its limitations. One major drawback is subjectivity: the lack of precise criteria for scoring ischemic changes can lead to variability among raters [23]. Furthermore, in patients with chronic ischemic changes, it can be difficult to differentiate acute events from preexisting lesions [23]. ASPECTS also does not adequately capture the location of specific occlusions (e.g., ICA, M1/M2 segments), often under-representing the involvement of the lenticulostriate branch and thus may underestimate the extent of ischemia [24,25]. ASPECTS also poorly reflects the true volume of the infarct, which is crucial in treatment decisions. On the contrary, CT perfusion (CTP) provides a more precise and volumetric assessment of the ischemic core and penumbra, helping to assess the viability of brain tissue. The literature supports the high reliability and consistency of CTP on various software platforms [24]. Furthermore, convolutional neural networks (CNNs) can generate clinically relevant perfusion maps from CT data while reducing radiation dose and improving accuracy [26]. In this context, HIR has been emphasised as a more precise, quantitative and objective marker than ASPECTS, especially for the selection of candidates for thrombectomy [27,28]. Therefore, this study prioritised the quantification of HIR and ischemic volume over the reliance on the ASPECTS.

Beyond HIR itself, collateral circulation plays a decisive role in extending the therapeutic window [29]. Collateral status is widely used to assess brain tissue viability and determine eligibility for thrombectomy. Studies show that good collateral flow significantly reduces final infarct volume, prolongs the therapeutic window, and improves outcomes—even in late-presenting patients [26,29]. These findings support the study hypothesis and reinforce collateral status as an essential independent prognostic factor. However, collateral classification remains largely qualitative, often relying on subjective scales such as the Tan scale, which depends on the experience of the assessor. Here, AI-based tools show potential; by integrating multiphase CTA with automated analysis, they may provide a more objective and reproducible assessment of collateral flow.

Integration of AI solutions into stroke diagnostics and treatment planning offers the potential to enhance diagnostic accuracy, personalise treatment strategies, and optimise time to intervention. AI algorithms capable of analysing perfusion maps, calculating HIR, and assessing collateral status can significantly reduce evaluation time and variability between readers [30]. This is especially valuable not only for tertiary stroke centres, but also for peripheral hospitals, where radiologist availability may be limited, potentially standardising diagnostic quality across healthcare settings. At the same time, several limitations must be acknowledged, including dependence on data quality, generalisability of trained algorithms, and challenges in interpretability [30]. These factors underscore the need for rigorous validation before AI tools are widely implemented in clinical practice.

This study has several limitations. First, its retrospective single-centre design is inherently subject to selection bias and may limit the generalisability of the findings. Second, the relatively small sample size may have reduced statistical power, particularly for secondary outcome analyses. Third, the cohort consisted predominantly of elderly patients with severe baseline neurological deficits, which may restrict the applicability of the results to other stroke populations. In addition, although a substantial part of the imaging analysis was based on automated software-derived parameters, the retrospective study design still limits full control over measurement conditions and data completeness.

## 5. Conclusions

In this single-centre retrospective cohort, lower hypoperfusion intensity ratio (HIR) values and better collateral status were associated with more favourable 90-day functional outcomes in patients with acute ischemic stroke treated with mechanical thrombectomy. In multivariable analysis, HIR remained an independent predictor alongside baseline NIHSS score and procedure duration, suggesting that HIR may provide complementary prognostic value in outcome assessment. Among automated CT perfusion parameters, infarct core volume and CBV index also demonstrated relevant predictive performance. These findings support the potential clinical utility of HIR as an imaging biomarker, although larger prospective studies are needed to confirm its role in routine practice.

## Figures and Tables

**Table 1:** Baseline demographic and clinical characteristics of the study population.

Variable	Value
Sex	
Male	36 (37.5%)
Female	60 (62.5%)
Patient age	75.8 ± 9.6
Clinical parameters	
NIHSS before treatment	16 (11.8–19.0)
mRS before treatment	5 (5.0–5.0)
mRS after 90 days	5 (1.8–6.0)
Primary arterial hypertension	76 (80%)
Intravenous thrombolysis before MT	
Yes	65 (67.7%)
No	31 (32.3%)
Final reperfusion grade (mTICI)	
0–2a	2 (2.1%)
2b–3	94 (97.9%)
Occlusion site	
Internal carotid artery (ICA)/carotid-T	30 (31.2%)
M1 segment	65 (67.7%)
M2 segment	1 (1.0%)
Anaesthesia strategy during MT	
General anaesthesia	15 (15.6%)
Conscious sedation/local	79 (82.3%)
Missing	2 (2.1%)
Diabetes mellitus (DM)	21 (22.1%)
Dyslipidemia	34 (35.8%)
Smoking	5 (5.3%)
History of transient ischemic attack (TIA)/cerebral infarction (CI)/intracerebral haemorrhage (ICH)	10 (10.5%)
Atrial fibrillation (AF)	66 (69.5%)
Coronary artery disease (CAD)/myocardial infarction (MI)	21 (22.1%)
Chronic heart failure (CHF)	43 (45.3%)
Deep vein thrombosis (DVT)/pulmonary artery thromboembolism (PTE)	2 (2.1%)

**Table 2:** Univariable associations and multivariable logistic regression models for predictors of poor functional outcome (mRS > 3 at 90 days).

Predictor	Univariable Association with Poor Outcome (mRS > 3)	Multivariable Logistic Regression—Model 1 (Without HIR) OR (95% CI)	p (Model 1)	Multivariable Logistic Regression—Model 2 (with HIR) OR (95% CI)	p (Model 2)
Procedure duration (per 1 min increase)	Shorter duration associated with favourable outcome (*p* < 0.001)	1.058 (1.018–1.099)	<0.005	1.051 (1.016–1.087)	<0.005
Baseline NIHSS (per 1 point increase)	Lower NIHSS in good outcome group (*p* = 0.004)	1.175 (1.020–1.353)	<0.05	1.184 (1.034–1.356)	<0.05
Baseline mRS (per 1 point)	Better pre-stroke mRS associated with favourable outcomes (*p* < 0.05)	– (not retained as an independent predictor in final model)			
Good collateral circulation (vs. poor/malignant)	Better collaterals associated with favourable outcome (*p* < 0.001)	0.086 (0.011–0.697)	<0.05	– (no longer significant and not retained in Model 2)	
Absence of diabetes mellitus (vs. presence)	Presence of diabetes associated with worse outcomes (*p* < 0.05)	0.131 (0.018–0.945)	<0.05	– (no longer significant and not retained in Model 2)	
HIR (per 0.1 unit increase)	Lower HIR in good outcome group (*p* < 0.001)	– (not included by definition)		1.476 (1.077–2.027)	<0.05

## Data Availability

The original contributions presented in the study are included in the article; further inquiries can be directed to the corresponding author.
